# Institutional student support and psychological well-being in Chinese vocational colleges: a two-institution cross-sectional study

**DOI:** 10.3389/fpsyg.2026.1944255

**Published:** 2026-10-15

**Authors:** Wang Hui, Huang Huifeng

**Affiliations:** 1 Student Affairs Department, Student Financial Assistance Center Fujian Forestry Vocational and Technical College, Nanping, Fujian, China; 2 Shuinan School, Nanping, Fujian, China

**Keywords:** Chinese higher education, psychological well-being, service quality, student support services, vocational education

## Abstract

Institutional student support is a potentially modifiable feature of the educational environment, but its relationship with distinct forms of positive psychological functioning remains underexamined in vocational higher education. This two-institution cross-sectional study examined associations between perceived quality of institutional student-support services and the six dimensions of Ryff’s psychological well-being among 383 students from public vocational colleges in Fujian Province, China. Students completed an 18-item Ryff-based well-being measure and a contextualized 50-item service-quality questionnaire covering accessibility, reliability, responsiveness, assurance, and empathy. Descriptive analyses addressed students’ well-being and service-quality profiles, and six multiple linear regression models examined associations between observed composite scores. Students reported comparatively stronger positive relations with others and purpose in life, whereas self-acceptance and personal growth were less favorable. Support services were generally rated favorably; accessibility was the lowest-rated service-quality domain and showed positive adjusted associations with all six well-being dimensions, whereas the other service-quality domains showed outcome-specific patterns. The models explained 53.3%–71.3% of outcome variance. These large R^2^ values should be interpreted cautiously because predictors and outcomes were collected through same-session self-report measures and may be affected by common-method variance or response-style effects. The findings identify service access and other service-quality domains as candidate priorities for institutional assessment, not as evidence that improving accessibility will directly improve well-being. Prospective, multi-source studies with validated measurement models are needed.

## 1 Introduction

Psychological well-being has become a central concern in higher education worldwide because students’ capacity to learn, persist, and participate productively depends on more than academic ability. Students must negotiate increasing independence, complex social relationships, performance pressure, uncertain employment prospects, and the administrative demands of institutional life (Chaudhry et al., 2024; Johnson et al., 2022). These demands are particularly salient in vocational and technical education, a sector that enrolls a substantial share of post-secondary students in many national systems and is closely linked to transitions into rapidly changing labor markets. Vocational programs are often intensive and employment-oriented, and their students frequently carry additional financial, family, and status-related pressures relative to peers in research universities. When students experience difficulty navigating these demands, academic engagement may weaken even when formal instruction is adequate. Conversely, when institutions provide dependable, approachable, and humane support, students may be better positioned to regulate effort, maintain purpose, seek help, and use available opportunities (Bakker and Mostert, 2024).

Contemporary accounts of student well-being distinguish positive functioning from the mere absence of psychological distress. Ryff’s eudaimonic model conceptualizes psychological well-being through six interrelated dimensions: self-acceptance, purpose in life, environmental mastery, positive relations with others, personal growth, and autonomy (Ryff, 1989, 2014; Ryff and Keyes, 1995). This framework is relevant to higher education because it captures the developmental work students undertake while learning. Self-acceptance concerns a balanced and affirmative view of oneself; purpose in life concerns direction and meaningful goals; environmental mastery reflects the capacity to manage circumstances and use opportunities; positive relations involve warm and trusting interpersonal ties; personal growth captures continuing development and openness to experience; and autonomy concerns self-directed judgment and action. These dimensions can vary within the same student population, yielding a profile that is more informative for institutional action than a single global well-being score.

Higher education institutions attempt to support student functioning through counseling, advising, registrar and records services, career assistance, grievance mechanisms, financial guidance, disability services, and digital service platforms. However, the existence of a service does not guarantee that students can use it effectively. Students may encounter unclear procedures, restricted office hours, slow responses, inconsistent information, fragmented referrals, or interactions that feel impersonal. International research on university support services therefore increasingly emphasizes the experienced quality of support rather than its nominal availability. Service utilization, responsiveness, and perceived institutional care are associated with adjustment, engagement, belonging, and help-seeking across diverse national contexts, although the magnitude and direction of these relationships vary (Barnes et al., 2024; Lawrie et al., 2025; McCabe et al., 2024; Wilson Fadiji and Eloff, 2024).

Related evidence also demonstrates why student well-being should be considered in context. A nationwide study in China reported different psychological-stress and sleep patterns among medical and non-medical students (Shen et al., 2025). In a separate 2-years study of severe COVID-19 survivors, loneliness, social relationships, and family communication during hospitalization were associated with later emotional distress (Romero-Moreno et al., 2025). Although these studies do not examine institutional support-service quality or Ryff’s dimensions among vocational students, they show that psychosocial factors and outcomes vary across populations and settings.

Service-quality theory offers a practical lens for examining students’ experiences of institutional support. The original SERVQUAL model distinguishes reliability, responsiveness, assurance, empathy, and tangibility (Parasuraman et al., 1988). The present study does not treat accessibility as a psychometric synonym for tangibility. Tangibility evaluates the visible physical evidence of service, such as facilities, equipment, and staff appearance; those features were not the focus of this inquiry, which examined whether students could actually reach and navigate student-support services delivered through both administrative offices and digital channels. Accessibility was therefore specified as a context-specific adaptation informed by higher-education service-quality research in which access is independently consequential (Abdullah, 2006). In this study, accessibility means functional reachability before and at entry to the service pathway: knowing where to seek help, locating clear service information, contacting personnel, using available physical or digital entry points, obtaining service at practicable times, understanding initial requirements, and being referred to the correct office without unnecessary confusion. This definition deliberately excludes post-contact response speed (responsiveness), consistency and fulfillment of commitments (reliability), competence and trustworthiness (assurance), and individualized care (empathy). The adaptation is therefore a SERVQUAL-informed contextualization for student services rather than a validated substitute for tangibility. Its proposed links with well-being are treated as plausible associations requiring further psychometric and longitudinal testing.

The Chinese higher education sector has expanded rapidly, and vocational colleges play a major role in preparing students for regional and national labor needs. Expansion has increased the diversity of student needs while intensifying academic, financial, and employment pressures. Studies involving Chinese university students have linked institutional or teacher support, belonging, social support, and service quality with thriving and well-being (Jiang et al., 2024; Wan et al., 2025; Yasmin et al., 2021; Zhang, 2024). Three gaps remain, however. First, much of the literature emphasizes psychological distress, general satisfaction, or a single support mechanism rather than the six dimensions of eudaimonic well-being. Second, student-support quality is often treated as a global construct, making it difficult to identify which operational service characteristics are associated with which developmental outcomes. Third, evidence specific to public vocational colleges in China remains limited despite the sector’s employment orientation, mass enrollment, and distinctive student-affairs demands. These gaps limit the ability of institutions to design service improvements that are both operationally targeted and developmentally relevant.

A domain-specific analysis is therefore important for both theory and practice. A service characteristic associated with students’ ability to manage their environment may not be the same characteristic associated with personal growth, purpose, or autonomy. Treating institutional support and psychological well-being as undifferentiated constructs can conceal potentially actionable patterns. By examining five operational service-quality domains alongside six Ryff well-being dimensions, the present study tests a more differentiated model of institutional support in vocational higher education.

The objective of this study was to determine how perceived quality of institutional student-support services is associated with multidimensional psychological well-being among students in two public vocational colleges in Fujian Province, China. The study addressed three research questions in a sequence mirrored in the sections “4 Results” and “5 Discussion”: (RQ1) What is the distribution of students across low, moderate, and high levels of the six dimensions of psychological well-being? (RQ2) How do students evaluate institutional student-support services in terms of accessibility, reliability, responsiveness, assurance, and empathy? and (RQ3) What are the joint and domain-specific statistical associations between the five service-quality domains and each psychological well-being dimension? The study expected the five service-quality domains to explain significant variance in each well-being outcome and the pattern of associations to differ across outcomes. The intended contribution is an empirically grounded framework for linking operational service characteristics to distinct forms of student functioning without implying causal effects from cross-sectional data.

## 2 Theoretical framework and hypotheses

### 2.1 Psychological well-being as a multidimensional outcome

Ryff’s model treats psychological well-being as a multidimensional pattern of positive functioning rather than a unitary emotional state (Ryff, 1989, 2014). In a college setting, self-acceptance may be challenged by academic comparison and transition-related uncertainty; purpose in life may be shaped by the perceived connection between current study and future work; environmental mastery may depend on students’ ability to navigate institutional systems; positive relations may draw from peer, faculty, and service interactions; personal growth may depend on opportunities for exploration and feedback; and autonomy may be strengthened when institutions provide information and respond to student voice. The six dimensions are conceptually related but not interchangeable, making separate outcome models analytically appropriate.

### 2.2 Service quality, accessibility, and institutional support as resources

The potential association between support quality and well-being can be understood through complementary theoretical perspectives. Study demands-resources theory proposes that institutional resources help students manage demands, sustain motivation, and protect well-being (Bakker and Mostert, 2024). Perceived organizational support theory suggests that people form judgments about whether an organization values them and cares about their welfare; these judgments are associated with affective and behavioral functioning (Eisenberger et al., 1986; Kurtessis et al., 2017). Self-determination theory adds a motivational account: environments that provide usable choices, acknowledge perspectives, and support competent action can facilitate autonomy and relatedness (Deci and Ryan, 2000). Together, these perspectives position student services as part of the educational environment through which institutions may reduce demands, signal care, enable agency, and provide relational support.

Within this integrated frame, the five service-quality domains have distinct operational meanings. Accessibility concerns functional reachability: identifying the appropriate service, locating or contacting it, entering through physical or digital channels, and navigating basic procedures at usable times. Reliability concerns consistency and fulfillment of commitments. Responsiveness concerns promptness after a request has been made and willingness to help. Assurance concerns competence, courtesy, confidentiality, and trustworthiness. Empathy concerns individualized attention and sensitivity to students’ circumstances. The boundaries are deliberately non-overlapping: for example, a service may be easy to reach (accessible) but slow after contact (not responsive), or highly competent (assured) but difficult to locate (not accessible). This conceptual separation is important because the study seeks to identify differentiated associations rather than a single global service-quality effect (Abdullah, 2006; Parasuraman et al., 1988).

### 2.3 Mechanisms linking service quality to psychological well-being

The proposed linkages between service quality and Ryff’s well-being dimensions are plausible mechanisms rather than established pathways. Accessibility may reduce administrative friction and uncertainty, creating conditions that support environmental mastery and autonomy and enabling students to benefit from other services. Reliability may strengthen predictability and continuity, which could support sustained goal pursuit and purpose in life. Responsiveness may reinforce agency by allowing students to make decisions with timely information. Assurance may provide an external scaffold of competence and trust that may be relevant to environmental mastery. Empathy may communicate relational affirmation and psychological safety, which could be associated with self-acceptance, positive relations, and personal growth. Related work with Spanish adolescents reported coping-related indirect effects in associations between emotional-intelligence dimensions and subjective well-being and perceived stress (Gil-Juliá et al., 2025), suggesting a candidate psychosocial pathway for future study. Coping was not measured here, and this work does not establish a mechanism for the present service-quality associations. These propositions motivate the differentiated analyses; the cross-sectional design tests contemporaneous statistical associations and cannot verify mechanisms, temporal ordering, or causal effects.

### 2.4 Hypotheses

The analysis specified one model-level hypothesis and one exploratory expectation. Hypothesis 1 (H1) stated that accessibility, reliability, responsiveness, assurance, and empathy would jointly explain statistically significant variance in each of the six psychological well-being dimensions. Hypothesis 2 (H2) was treated as an exploratory expectation that the pattern of unique associations would vary across the six outcomes. H2 was not interpreted as a claim that coefficients differed statistically across separate regression equations; such a claim would require formal cross-model coefficient comparisons, for example through a multivariate or seemingly unrelated regression framework. The hypotheses concern associations among observed composite scores and do not constitute claims about latent structural paths or causal effects.

## 3 Materials and methods

### 3.1 Research design

The study used a quantitative, two-institution, cross-sectional correlational design. Students completed self-report measures of psychological well-being and perceived institutional student-support services at one point in time. The first two research questions were descriptive; the third was associational. Multiple linear regression was used to model each observed well-being composite score as a function of the five observed service-quality composite scores. Regression was therefore not used as a latent-variable model. A structural equation model would be required to estimate measurement-error-adjusted relations among latent constructs; that was beyond the measurement evidence and aims of the present study. The design is appropriate for identifying contemporaneous statistical associations, but it cannot establish temporal order or causality.

### 3.2 Setting and participants

The study was conducted at Fujian Forestry Vocational and Technical College and Minbei Vocational and Technical College in Fujian Province, China. Their combined enrollment was 26,383 students (17,383 and 9,000, respectively). A proportionate institutional allocation set a planned minimum of 379 students (250 and 129, respectively). Because the individual sampling frame and selection procedure were not documented, the sample is described as institutionally proportionate rather than probability-based. The final analysis included 383 complete questionnaires. Institution-specific analyzed counts, response rates, and distributions of age, gender, year level, and academic program were not reported. These limitations restrict assessment of selection bias and subgroup representation. Table 1 summarizes the institutional population and planned allocation.

**TABLE 1:** Institutional population and planned allocation.

Institution	Population	Planned allocation (*n*)
Fujian Forestry Vocational and Technical College	17,383	250
Minbei Vocational and Technical College	9,000	129
Total	26,383	379

### 3.3 Measures

Psychological well-being was assessed using an 18-item short form based on Ryff’s multidimensional model, with three items representing each dimension: self-acceptance, purpose in life, environmental mastery, positive relations with others, personal growth, and autonomy (Ryff and Keyes, 1995). Responses were recorded on a 7-point agreement scale ranging from 1 (strongly disagree) to 7 (strongly agree). Negatively worded items were reverse-scored before the three items in each dimension were summed, yielding a possible score of 3–21. For descriptive interpretation, scores of 3–8 were classified as low, 9–14 as moderate, and 15–21 as high. These bands were specified for this study rather than taken as validated clinical or normative cutoffs. The short form permits efficient multidimensional assessment but provides less measurement breadth than longer Ryff versions; prior psychometric work has also shown that the factor structure of shortened Ryff measures requires cautious interpretation (Abbott et al., 2006). The analysis provided only the total 18-item Cronbach’s alpha (α = 0.98); separate reliability coefficients for the six three-item subscales were not available. The very high total alpha is interpreted cautiously because it may reflect item redundancy, response style, or overlap among dimensions; it is not treated as evidence of a six-factor latent structure.

Perceived service quality was measured using a contextualized, researcher-developed 50-item questionnaire, with 10 items for each domain: accessibility, reliability, responsiveness, assurance, and empathy. The instrument was SERVQUAL-informed rather than a direct reproduction of SERVQUAL. Reliability, responsiveness, assurance, and empathy retain their established service-quality meanings (Parasuraman et al., 1988), whereas accessibility was specified as a higher-education service-quality domain because the study focused on functional reachability rather than on physical tangibles. The 10 accessibility items operationalized four closely related facets of reachability: service discoverability, contactability and temporal availability, channel/procedural navigability, and referral/communication access. These items were designed not to duplicate post-contact promptness, consistency, staff competence, or individualized care, which were measured under responsiveness, reliability, assurance, and empathy, respectively. Items were rated on a 5-point scale from 1 (very low extent) to 5 (very great extent). For descriptive interpretation, the study used the following bands: 1.00–1.79 (very low), 1.80–2.59 (low), 2.60–3.39 (moderate), 3.40–4.19 (great), and 4.20–5.00 (very great extent). Instrument development included domain specification, item drafting for the vocational-college context, expert content review, revision based on expert comments, pilot testing with students outside the final sample, internal-consistency assessment, and translation/back-translation where needed. These procedures provide content, face, linguistic, and reliability evidence but do not establish the five-domain latent structure (Boateng et al., 2018). No exploratory or confirmatory factor analysis or five-domain correlation matrix was available. The five-domain structure is therefore provisional, and the service-quality domains are analyzed as theoretically specified observed composite scores.

### 3.4 Procedure and ethics

Ethical approval was obtained from Batangas State University, The National Engineering University, and institutional permission was obtained from the participating Chinese colleges before questionnaire administration. The approval reference number and date, as well as the responsible offices and reference details for the China-side permissions or local ethics review, were not available for inclusion in the article. Participants were informed of the study purpose, procedures, voluntary nature of participation, confidentiality protections, intended use of the data, and their right to withdraw without penalty. Written informed consent was obtained before participation. Completed questionnaires were checked for completeness, encoded for analysis, and handled without personally identifying information in the analytic file or reported results.

### 3.5 Data analysis

The analytic strategy followed the three research questions. For RQ1, frequencies and percentages summarized low, moderate, and high descriptive bands for each psychological well-being dimension. For RQ2, service-quality domain means summarized students’ evaluations of accessibility, reliability, responsiveness, assurance, and empathy. For RQ3, six simultaneous multiple linear regression models were estimated, one for each psychological well-being dimension, with the composite score for each service-quality domain entered together. The regression models therefore analyze observed composite scores; they do not estimate latent constructs or measurement-error-adjusted structural paths. R^2^, adjusted R^2^, and F statistics summarize the joint association of the five service-quality domains with each well-being outcome, while unstandardized coefficients (B), t statistics, and *p*-values summarize the unique association of each service-quality domain conditional on the others. Because all five service predictors were measured on the same 1–5 response metric, within-model comparisons of unstandardized coefficients are descriptive comparisons of slope magnitude, not causal effect estimates. Statistical significance was evaluated at α = 0.05; values reported as 0.000 in the regression output are presented as *p* < 0.001. For coefficient-level inference, the 30 predictor tests were treated as exploratory and approximate Holm-adjusted *p*-values are shown in Table 5. Standard errors and 95% confidence intervals in Table 5 were reconstructed from the reported B and *t*-values with df = 377. Because the regression summary provided rounded values, the reconstructed values and Holm-adjusted *p*-values should be considered approximate. The regression output did not include standardized coefficients, predictor correlations, VIF/tolerance, residual diagnostics, influential-observation statistics, an institution-adjusted sensitivity analysis, or a regression-based power analysis. The original sample-size calculation is therefore interpreted as a recruitment target rather than as a regression-based power justification.

## 4 Results

### 4.1 RQ1: psychological well-being profile

RQ1 examined the distribution of psychological well-being across the six Ryff dimensions. As shown in Table 2, positive relations with others was the strongest dimension: 206 students (53.8%) were classified at the high level and none at the low level. Purpose in life was also comparatively favorable, with 141 students (36.8%) at the high level and 193 (50.4%) at the moderate level. Environmental mastery and autonomy were concentrated at the moderate level, representing 56.4% and 52.2% of respondents, respectively.

**TABLE 2:** Distribution of psychological well-being levels (*N* = 383).

Dimension	Low *n* (%)	Moderate *n* (%)	High *n* (%)
Self-acceptance	241 (62.9)	142 (37.1)	0 (0.0)
Purpose in life	49 (12.8)	193 (50.4)	141 (36.8)
Environmental mastery	135 (35.2)	216 (56.4)	32 (8.4)
Positive relations	0 (0.0)	177 (46.2)	206 (53.8)
Personal growth	207 (54.0)	176 (46.0)	0 (0.0)
Autonomy	158 (41.3)	200 (52.2)	25 (6.5)

Each dimension comprised three items scored from 1 to 7. Low = 3–8; moderate = 9–14; high = 15–21. These are author-defined, approximately equal-width descriptive bands across the possible score range; they are not clinical cutoffs or validated normative categories and should not be interpreted as diagnostic levels.

In contrast, self-acceptance and personal growth were the weakest dimensions. For self-acceptance, 241 students (62.9%) were classified at the low level and no respondent reached the high category. For personal growth, 207 students (54.0%) were at the low level and again no respondent reached the high category. These descriptive results answer RQ1 by showing a differentiated rather than uniformly high or low well-being profile.

### 4.2 RQ2: perceived quality of institutional student-support services

RQ2 examined students’ evaluations of the five service-quality domains. As shown in Table 3, assurance received the highest composite mean (*M* = 3.57), followed by empathy (*M* = 3.55), responsiveness (*M* = 3.53), and reliability (*M* = 3.51); each was interpreted as a great extent. Accessibility received the lowest mean (*M* = 3.42), which also fell within the great-extent band but was comparatively weaker than the other service-quality domains. Thus, RQ2 indicates that students generally viewed the quality of support positively while reporting relatively greater difficulty at the point of service access.

**TABLE 3:** Perceived quality of institutional student-support services (*N* = 383).

Service-quality domain	M	Interpretation
Accessibility	3.42	Great extent
Reliability	3.51	Great extent
Responsiveness	3.53	Great extent
Assurance	3.57	Great extent
Empathy	3.55	Great extent

Scale range: 1–5. Very low extent = 1.00–1.79; low extent = 1.80–2.59; moderate extent = 2.60–3.39; great extent = 3.40–4.19; very great extent = 4.20–5.00.

### 4.3 RQ3: associations between service quality and psychological well-being

RQ3 examined the joint and domain-specific statistical associations between the five service-quality domains and each well-being outcome. At the model level, all six regression equations were statistically significant (Table 4). The five service-quality domains jointly accounted for 71.3% of the variance in self-acceptance, 69.6% in purpose in life, 63.7% in environmental mastery, 53.3% in positive relations with others, 68.3% in personal growth, and 65.4% in autonomy. These values indicate strong contemporaneous associations in the sampled institutions; they should not be interpreted as causal effects. Their magnitude may be partly influenced by common-method variance because predictors and outcomes were collected in the same self-report session. The model-level R^2^ values also do not establish that service quality explains the corresponding well-being dimensions.

**TABLE 4:** Summary of regression models for psychological well-being dimensions.

Outcome	R^2^	Adj. R^2^	*F* (5, 377)	*p*
Self-acceptance	0.713	0.709	187.59	<0.001
Purpose in life	0.696	0.692	172.34	<0.001
Environmental mastery	0.637	0.632	132.38	<0.001
Positive relations	0.533	0.527	86.00	<0.001
Personal growth	0.683	0.679	162.79	<0.001
Autonomy	0.654	0.649	142.35	<0.001

Explanatory variables in each model: accessibility, reliability, responsiveness, assurance, and empathy. Adjusted R^2^ values are calculated from the reported R^2^ values using *N* = 383 and five explanatory variables.

#### 4.3.1 Domain-specific associations

Accessibility was the only service-quality domain with a positive coefficient that was statistically significant in all six models at the unadjusted and approximate Holm-adjusted levels (Table 5). The association was positive for self-acceptance (*B* = 4.153, *t* = 9.765, *p* < 0.001), purpose in life (*B* = 3.672, *t* = 7.862, *p* < 0.001), environmental mastery (*B* = 2.531, *t* = 5.021, *p* < 0.001), positive relations with others (*B* = 3.723, *t* = 6.137, *p* < 0.001), personal growth (*B* = 2.209, *t* = 4.725, *p* < 0.001), and autonomy (*B* = 2.257, *t* = 4.528, *p* < 0.001). Because all service predictors use the same response metric, the coefficients can be compared descriptively within each outcome model; however, they remain cross-sectional slopes rather than causal effects. Standardized coefficients were not available in the regression output; therefore, Table 5 reports unstandardized coefficients.

**TABLE 5:** Regression coefficients for service-quality associations.

Outcome/service-quality domain	B	SE^†^	95% CI^†^	*t*	*p*	pHolm^†^
Self-acceptance						
Accessibility	4.153	0.425	[3.317, 4.989]	9.765	<0.001	<0.001
Reliability	1.670	0.394	[0.895, 2.445]	4.237	<0.001	<0.001
Responsiveness	1.936	0.391	[1.167, 2.705]	4.952	<0.001	<0.001
Assurance	0.926	0.380	[0.179, 1.673]	2.436	0.015	0.168
Empathy	1.635	0.373	[0.902, 2.368]	4.384	<0.001	<0.001
Purpose in life						
Accessibility	3.672	0.467	[2.754, 4.590]	7.862	<0.001	<0.001
Reliability	4.009	0.433	[3.158, 4.860]	9.261	<0.001	<0.001
Responsiveness	1.088	0.430	[0.243, 1.933]	2.531	0.012	0.141
Assurance	0.475	0.417	[−0.346, 1.296]	1.138	0.256	1.000
Empathy	1.183	0.410	[0.378, 1.988]	2.888	0.004	0.053
Environmental mastery						
Accessibility	2.531	0.504	[1.540, 3.522]	5.021	<0.001	<0.001
Reliability	0.852	0.467	[−0.066, 1.770]	1.824	0.069	0.483
Responsiveness	0.558	0.464	[−0.355, 1.471]	1.202	0.230	1.000
Assurance	5.232	0.450	[4.346, 6.118]	11.616	<0.001	<0.001
Empathy	1.330	0.442	[0.461, 2.199]	3.011	0.003	0.042
Positive relations with others						
Accessibility	3.723	0.607	[2.530, 4.916]	6.137	<0.001	<0.001
Reliability	2.107	0.562	[1.002, 3.212]	3.748	<0.001	0.004
Responsiveness	1.322	0.559	[0.224, 2.420]	2.367	0.018	0.184
Assurance	1.106	0.542	[0.040, 2.172]	2.040	0.042	0.378
Empathy	1.739	0.532	[0.693, 2.785]	3.270	0.001	0.019
Personal growth						
Accessibility	2.209	0.468	[1.290, 3.128]	4.725	<0.001	<0.001
Reliability	1.264	0.433	[0.412, 2.116]	2.918	0.004	0.052
Responsiveness	0.546	0.430	[−0.300, 1.392]	1.269	0.205	1.000
Assurance	0.568	0.417	[−0.253, 1.389]	1.361	0.174	1.000
Empathy	5.936	0.410	[5.130, 6.742]	14.483	<0.001	<0.001
Autonomy						
Accessibility	2.257	0.498	[1.277, 3.237]	4.528	<0.001	<0.001
Reliability	0.640	0.462	[−0.269, 1.549]	1.385	0.167	1.000
Responsiveness	6.093	0.459	[5.191, 6.995]	13.280	<0.001	<0.001
Assurance	0.450	0.445	[−0.425, 1.325]	1.011	0.313	1.000
Empathy	0.830	0.437	[−0.030, 1.690]	1.898	0.058	0.468

B, unstandardized regression coefficient; SE, standard error; CI, confidence interval; pHolm, approximate Holm-adjusted *p*-value across the 30 coefficient-level tests. Each model includes accessibility, reliability, responsiveness, assurance, and empathy simultaneously. SE and 95% CIs were reconstructed from rounded B and *t*-values with df = 377. pHolm values were calculated from rounded *t* statistics and should be treated as approximate. Standardized coefficients, predictor correlations, and regression diagnostics were not available in the regression output.

† Denotes approximate values derived from rounded regression statistics.

The remaining service-quality domains showed outcome-specific patterns. Before multiplicity adjustment, reliability had the largest reported coefficient for purpose in life (*B* = 4.009, *t* = 9.261, *p* < 0.001), assurance had the largest coefficient for environmental mastery (*B* = 5.232, *t* = 11.616, *p* < 0.001), empathy had the largest coefficient for personal growth (*B* = 5.936, *t* = 14.483, *p* < 0.001), and responsiveness had the largest coefficient for autonomy (*B* = 6.093, *t* = 13.280, *p* < 0.001). After approximate Holm adjustment, reliability remained significant for self-acceptance, purpose in life, and positive relations; assurance remained significant for environmental mastery; empathy remained significant for self-acceptance, environmental mastery, positive relations, and personal growth; and responsiveness remained significant for self-acceptance and autonomy. These adjusted results are exploratory because the *p*-values were reconstructed from rounded *t* statistics. They also do not constitute formal tests that coefficients differ across outcomes.

At the level of observed composite scores, H1 was supported at the model level because the five service-quality domains jointly explained statistically significant variance in every well-being dimension. The observed pattern was consistent with the exploratory expectation represented by H2, but H2 was not formally tested as a difference between coefficients across separate outcome models; it is therefore not described as statistically confirmed. The regression output did not include VIF or tolerance, predictor correlations, standardized coefficients, residual-assumption checks, influential-observation diagnostics, or an institution-sensitivity analysis. These omissions are reported because they affect interpretation of the regression estimates and limit the strength of the conclusions.

## 5 Discussion

The discussion follows the sequence of the research questions. RQ1 showed a differentiated well-being profile, with comparatively stronger positive relations with others and purpose in life but weaker self-acceptance and personal growth. RQ2 showed generally favorable evaluations of support services alongside a relative weakness in accessibility. RQ3 showed strong statistical associations between service-quality composites and well-being composites, with accessibility associated with every outcome and other service-quality domains displaying more differentiated patterns. These patterns describe the sampled students’ contemporaneous responses; they do not demonstrate that any service feature causes or improves a well-being outcome.

### 5.1 RQ1: relational strengths and developmental vulnerabilities

The high level of positive relations suggests that many students experienced meaningful social connection. Belonging and supportive relationships are consistently associated with psychological and academic adjustment in higher education (Lawrie et al., 2025; Romeo et al., 2024). Strong peer networks may be especially important in vocational colleges, where students often progress through structured cohorts and practical learning activities. The relatively favorable purpose-in-life scores may likewise reflect students’ orientation toward specific occupations and the applied nature of vocational education. Clear links between coursework and employment can provide direction even when other aspects of well-being remain fragile.

The weak self-acceptance and personal-growth scores require particular attention. Students may be able to maintain relationships and pursue externally defined goals while still doubting their own worth, capabilities, or developmental progress. Academic comparison, pressure to secure employment, family expectations, and uncertainty about the societal status of vocational pathways may contribute to these vulnerabilities. Wu et al. (2024) found that self-acceptance and social support occupy important positions in the network of meaning in life among college students, while broader evidence indicates that supportive, strengths-oriented interventions can improve student well-being (Abulfaraj et al., 2024; Worsley et al., 2022). The present profile therefore argues against relying exclusively on crisis counseling. Preventive programming should also help students recognize strengths, interpret setbacks constructively, and identify evidence of personal development.

### 5.2 RQ2: service quality and the accessibility gap

Accessibility was the lowest-rated service-quality domain while also showing significant associations with all six well-being dimensions. We refer to this descriptive combination as an accessibility gap rather than as evidence of a causal mechanism. In higher-education service research, access has been identified as a distinct service-quality domain (Abdullah, 2006), and research on student support shows that nominal availability is insufficient when students cannot identify, reach, or navigate appropriate services (Barnes et al., 2024; Johnson et al., 2022; McCabe et al., 2024). Students may therefore evaluate staff competence and empathy positively once contact occurs while still experiencing friction at the entry stage, such as unclear office functions, restricted schedules, fragmented digital channels, or uncertainty about where to seek help.

One plausible interpretation is that accessibility has broad associations because it functions as an entry condition for other service experiences. If students cannot locate or enter a service pathway, they have fewer opportunities to experience its reliability, responsiveness, assurance, or empathy. Accessible systems may also reduce administrative uncertainty and conserve cognitive and emotional resources. In study demands-resources terms, clear and reachable support can be conceptualized as an institutional resource for managing demands (Bakker and Mostert, 2024); from an organizational-support perspective, ease of access may also contribute to perceptions that institutional concern is genuine rather than merely symbolic (Kurtessis et al., 2017). These are theoretical interpretations of the observed associations, not evidence that improving accessibility will directly improve well-being. They require longitudinal, multi-source, or experimental testing.

### 5.3 RQ3: domain-specific associations and plausible mechanisms

The association patterns are consistent with the conceptual framework in Figure 1, although the cross-sectional design cannot verify the proposed mechanisms. Reliability showed its largest reported coefficient for purpose in life. Dependable procedures and consistent follow-through may reduce uncertainty and help students sustain goal-directed planning, a possibility consistent with a resource-based interpretation of institutional support (Bakker and Mostert, 2024). This should be interpreted as a plausible explanation for the observed association rather than evidence that reliability produces purpose in life.

**FIGURE 1:**
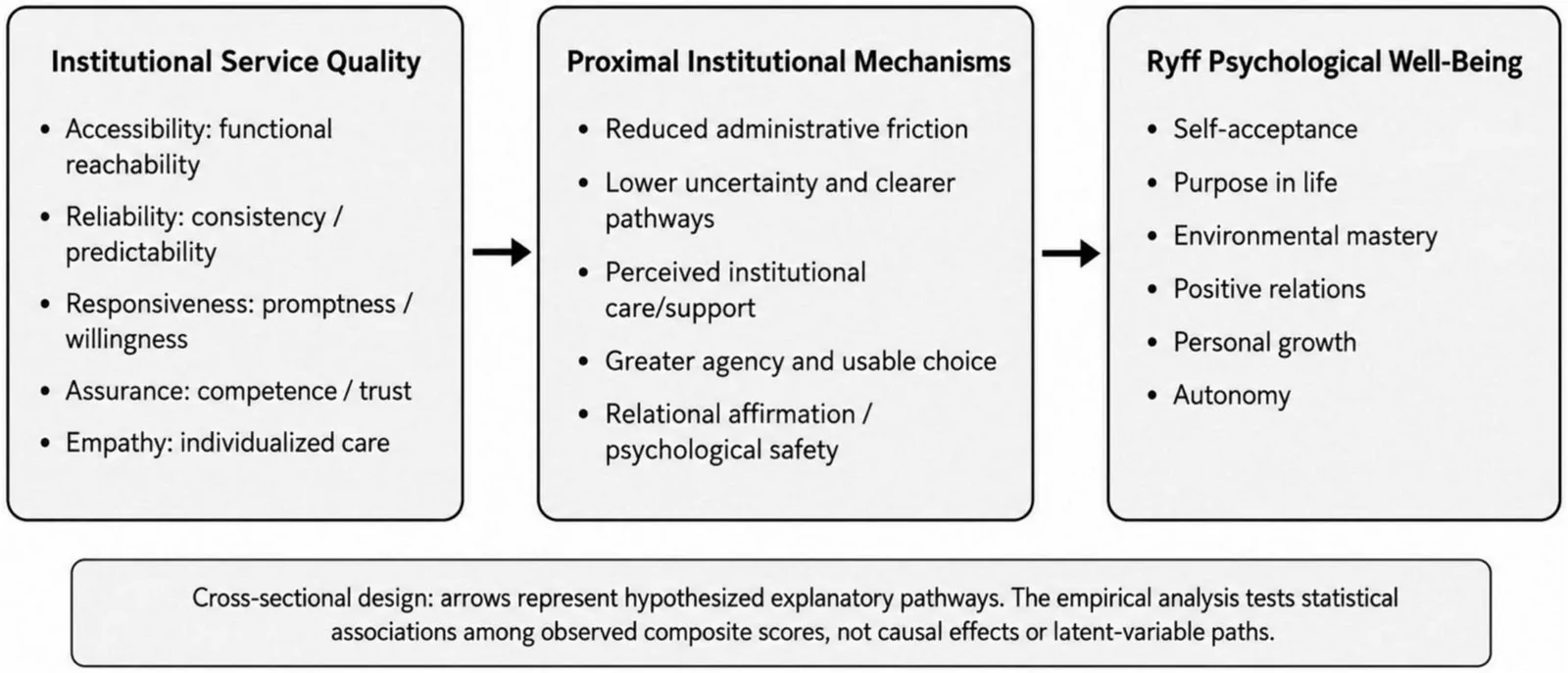
Conceptual framework linking the five institutional student-support service-quality domains, plausible proximal mechanisms, and Ryff’s six psychological well-being dimensions. Arrows denote hypothesized explanatory pathways rather than demonstrated causal effects.

Assurance showed its largest reported coefficient for environmental mastery. Staff competence, confidentiality, and trustworthiness may provide a credible external resource when students navigate academic, financial, or administrative problems. Because environmental mastery concerns the effective use of opportunities and management of one’s surroundings (Ryff, 2014), confidence in knowledgeable personnel is theoretically congruent with this outcome. The nonsignificance of reliability and responsiveness in this model does not imply that those domains are operationally unimportant; it indicates only that their unique statistical associations were not significant after the other service-quality domains were entered simultaneously.

Empathy showed the largest reported coefficient for personal growth and was also associated with several relational or self-evaluative outcomes. Individualized, nonjudgmental support may provide relational affirmation and psychological safety for reflection and development. This interpretation is consistent with evidence linking social support and person-centered institutional experiences with student well-being (Li et al., 2025; Wilson Fadiji and Eloff, 2024), but the present data cannot establish mediation or temporal order.

Responsiveness showed the largest reported coefficient for autonomy. Timely institutional action may enable students to make decisions with current information and maintain a sense of agency. This pattern is theoretically consistent with self-determination theory, in which autonomy is supported when environments acknowledge perspectives and enable meaningful choice (Deci and Ryan, 2000). Again, the result is an association among contemporaneously measured composites rather than a demonstrated causal pathway.

### 5.4 Implications for practice

The findings motivate a tiered strategy for student services as a practical recommendation for institutional assessment, not as an intervention demonstrated to be effective by this study. The first tier should address universal access: a single, well-publicized service directory; clear descriptions of office responsibilities; consistent physical and digital access points; transparent requirements and expected processing times; accessible appointment systems; and referral protocols that prevent students from being passed repeatedly between offices. Information should be available in formats suitable for mobile access and for students with disabilities. Institutions should routinely examine whether opening hours, platform design, and communication practices match students’ actual schedules and capabilities.

The second tier should strengthen the human quality of service encounters. Staff development should combine procedural knowledge with communication, confidentiality, cultural sensitivity, and psychologically safe interaction. Empathy should be treated as an observable professional practice rather than a personality trait: listening without premature judgment, explaining decisions, recognizing individual constraints, and following up when a student’s concern involves multiple offices. Quality monitoring should therefore include both efficiency indicators and student-reported experience.

The third tier should connect services to developmental outcomes. Because self-acceptance and personal growth were less favorable in this sample, colleges could evaluate strengths-based advising, reflective career planning, peer mentoring, and guided goal review as possible supports. Advisers and student-affairs personnel can help students identify progress, reframe setbacks, and connect competencies to future roles. Purpose-oriented support could emphasize reliable follow-through and clear pathways from study to employment. Autonomy-supportive services could provide options, explain rationales, and respond to student requests within defined timelines. Environmental-mastery initiatives could ensure that students know how to mobilize credible expertise when navigating academic, financial, or personal problems. These are testable recommendations, not effects demonstrated by the present cross-sectional study.

The results also have implications for faculty and educational leaders. Although the measures focused on institutional student-support services rather than classroom teaching, students encounter institutional support as part of the broader learning environment. Faculty advisers are often the first to notice difficulty and the first referral point to formal services. Effective support therefore requires coordination among teachers, advisers, counselors, administrators, and digital-service teams. Referral literacy, clear role boundaries, and feedback loops can prevent students from becoming lost between academic and administrative systems. In vocational colleges, coordination is especially important because internships, certifications, and employment transitions frequently involve multiple institutional units.

### 5.5 Theoretical contribution and interpretive cautions

The study’s theoretical contribution is to connect operational service-quality domains with multiple forms of eudaimonic well-being rather than with satisfaction alone. It also clarifies that accessibility is a context-specific functional-reachability construct, not a synonymous replacement for SERVQUAL tangibility. The observed pattern is compatible with an integrated resource model: accessibility may provide a common entry condition, while reliability, responsiveness, assurance, and empathy may have different psychological affordances. This interpretation extends higher-education service-quality work that identifies access as a meaningful service-quality domain (Abdullah, 2006) and aligns it with Ryff’s multidimensional account of positive functioning (Ryff, 1989, 2014). Because construct validity of the study-specific service instrument has not yet been established by factor analysis, this theoretical contribution should be treated as provisional and tested in future measurement models (Boateng et al., 2018).

The large R^2^ values warrant caution as well as interest. Predictors and outcomes were collected from the same respondents in the same session, so common-method variance and response-style effects may have inflated associations (Podsakoff et al., 2003). In addition, unstandardized coefficients are scale-dependent and should not be interpreted as causal effect sizes. Replication with longitudinal designs, multiple data sources, standardized effect estimates, and psychometrically confirmed measurement models is needed before strong claims are made about comparative influence.

### 5.6 Implications for institutional assessment

A meaningful response to these findings requires more than a one-time action plan. Colleges should establish an assessment cycle in which student-service data are collected, reviewed, acted upon, and reassessed. At the beginning of each academic year, institutions can map the complete student journey–from initial inquiry and enrollment to advising, internships, graduation, and career transition–and identify points at which students must seek information or approval. For each point, administrators can specify responsible offices, required documents, expected response times, escalation procedures, and accessible alternatives. This mapping makes hidden administrative demands visible, provides a basis for reducing unnecessary steps, and helps distinguish problems of capacity from problems of communication, coordination, or service design.

Assessment indicators should combine operational performance with developmental outcomes. Operational indicators may include waiting time, response time, referral completion, repeated visits for the same concern, platform downtime, unresolved cases, and use by different student groups. Experience indicators may include perceived clarity, fairness, confidentiality, respect, and confidence in the guidance received. Developmental indicators can track whether students report greater self-acceptance, personal growth, purpose, mastery, relatedness, and autonomy after targeted initiatives. Because the present findings are correlational, institutions should introduce improvements in stages and evaluate change with pre- and post-intervention measures, comparison groups where feasible, and qualitative feedback that clarifies why an initiative did or did not work.

Equity should be built into this assessment cycle. A satisfactory average can conceal barriers experienced by students with disabilities, limited financial resources, rural backgrounds, language needs, family responsibilities, or low familiarity with institutional procedures. Service dashboards should therefore disaggregate use and experience data where ethically and statistically appropriate. Institutions should also provide confidential mechanisms through which students can report inaccessible or disrespectful encounters without fear of academic consequences, and co-design sessions with students can identify procedural burdens that are obvious to service users but invisible to administrators.

## 6 Limitations and future research

Several limitations constrain interpretation. First, the cross-sectional design cannot establish whether better support promotes well-being, whether students with higher well-being perceive services more positively, or whether both are influenced by unmeasured factors. Longitudinal and intervention studies are needed to examine temporal ordering. Second, all variables were self-reported in the same data-collection session, creating potential common-method bias, social-desirability effects, and response-style artifacts (Podsakoff et al., 2003). Future studies should combine surveys with service-use records, processing-time data, qualitative interviews, and staff perspectives.

Third, the sample was drawn from two public vocational colleges in one Chinese province. The two-institution design provides evidence across more than one organizational setting but does not support broad generalization to research universities, private institutions, other provinces, or other countries. Institution-specific analyzed counts, response rates, and basic demographic and academic characteristics were not reported; these omissions prevent evaluation of selection bias and subgroup differences. Demographic and academic characteristics were not included in the article-level analysis, preventing tests of whether associations differed by gender, year level, program, socioeconomic position, residence status, or prior service use. The small number of institutions also prevents reliable estimation of institution-level effects; future multi-site studies should include enough institutions to permit cluster-robust or multilevel analysis.

Fourth, measurement evidence is incomplete. The 18-item well-being measure uses only three items per dimension, and the researcher-developed service instrument has evidence of content review, translation/back-translation, and internal consistency but not exploratory or confirmatory factor analysis, convergent validity, discriminant validity, or measurement invariance. Cronbach’s alpha alone is not evidence of construct validity (Boateng et al., 2018). The very high overall homogeneity of the well-being measure may also reflect item redundancy or response-style artifacts. Future research should conduct item-level psychometric evaluation, confirm the factor structures, assess convergent and discriminant validity, and test invariance across institutions and demographic groups. Fifth, the regression output did not include standardized coefficients, predictor correlations, VIF/tolerance, residual-assumption checks, or influential-observation statistics. The reconstructed SE and CIs in Table 5 do not substitute for these diagnostics. Students were nested within institutions, but the analysis did not model institutional clustering or provide an institution-adjusted sensitivity analysis; future studies should use multilevel models or cluster-robust standard errors when the number and structure of institutions permit.

Finally, the study examined institutional support services but did not directly measure teaching practice or classroom-level support. Future research could integrate teacher variables–such as autonomy-supportive teaching, faculty referral practices, and advising quality–to examine how academic and administrative support systems jointly shape student well-being.

## 7 Conclusion

Among students from the two participating public vocational colleges in Fujian Province, China, positive relations with others and purpose in life were comparatively stronger, whereas self-acceptance and personal growth were less favorable. Students generally evaluated institutional support services favorably, but accessibility received the lowest rating among the five service-quality domains and was positively associated with all six psychological well-being dimensions in the adjusted models. Reliability, assurance, empathy, and responsiveness showed different observed association patterns across outcomes. These findings support assessing functional access as a distinct, context-specific service-quality domain in higher education (Abdullah, 2006) and examining its association with Ryff’s multidimensional conception of psychological well-being (Ryff, 1989, 2014). They reflect the self-reported experiences of students at these two institutions and should not be generalized to other colleges without further evidence. Institutions may evaluate functional access while maintaining dependable, timely, trustworthy, and person-centered services; the study does not show that such changes will improve well-being. Because the evidence is cross-sectional and self-reported, the service questionnaire lacks factor-analytic validation, and key regression diagnostics were unavailable, the findings identify institutional priorities and testable pathways rather than causal effects.

## Data Availability

The datasets presented in this study can be found in online repositories. The names of the repository/repositories and accession number(s) can be found in the article/supplementary material.
